# Uterine tumors resembling ovarian sex cord tumors: rare case report and literature review

**DOI:** 10.3389/fonc.2026.1779513

**Published:** 2026-02-16

**Authors:** Kaige Pei, Xiumei Xu, Yonghong Li, Yuhao Long, Mingrong Xi, Ruiqi Duan

**Affiliations:** 1 Department of Obstetrics and Gynecology, West China Second University Hospital, Sichuan University, Chengdu, China; 2 Key Laboratory of Birth Defects and Related Diseases of Women and Children (Sichuan University), Ministry of Education, Chengdu, China; 3 Department of Obstetrics and Gynecology, The People’s Hospital of Wenjiang District, Chengdu, China; 4 Department of Obstetrics and Gynecology, Meishan Women and Children’s Hospital, Alliance Hospital of West China Second University Hospital, Sichuan University, Meishan, China

**Keywords:** fertility-sparing surgery, literature review, molecular classification, rare case report, UTROSCT

## Abstract

Uterine tumors resembling ovarian sex-cord tumors (UTROSCT) are rare mesenchymal neoplasms with low malignant potential and polyphenotypic immunoprofiles. A 38-year-old nullipara presented with menorrhagia; trans-vaginal ultrasound revealed a 26-mm, richly vascular submucosal mass. Complete hysteroscopic excision was achieved without residual disease. Microscopy showed anastomosing cords, hollow tubules and bland spindled cells (0–1 mitosis/10 HPF). Immunohistochemistry demonstrated diffuse AE1/3, CD56, WT-1 and synaptophysin positivity, while inhibin-α, calretinin and ER/PR were negative; desmin highlighted entrapped myometrium. Ki-67 index was ~5%. Break-apart FISH and next-generation sequencing (141-gene solid-tumor panel) disclosed no pathogenic fusions involving ESR1, GREB1, NCOA2/3 or NR4A3, nor mutations in TP53, BRCA1/2 or mismatch-repair genes, consistent with the fusion-negative UTROSCT subset (~30–45% of cases). At 20 months the patient is disease-free with regular menses and intact fertility. We review diagnostic clues, differential diagnoses, molecular taxonomy and fertility-sparing strategies, underscoring the value of comprehensive genomic profiling for accurate classification and prognostication of this uncommon uterine tumor.

## 1 Introduction

Uterine tumors resembling ovarian sex cord tumors (UTROSCTs) represent a rare and diagnostically challenging group of mesenchymal neoplasms with uncertain malignant potential (1–3). First described in 1945, these tumors have remained an enigma in gynecologic pathology due to their histogenetic ambiguity and variable clinical behavior (4, 5). UTROSCTs typically occur in peri-menopausal and postmenopausal women, with a mean age at diagnosis of approximately 52 years, though cases have been reported across a wide age spectrum from 19 to 86 years (2, 6, 7). The clinical presentation is often non-specific, with abnormal vaginal bleeding being the most common symptom, followed by pelvic pain, uterine enlargement, or incidental discovery during imaging studies (8–10). Approximately 20% of patients are asymptomatic at diagnosis (7). The histogenesis of UTROSCTs remains controversial, with theories suggesting origin from pluripotent uterine mesenchymal cells or endometrial stromal cells with secondary sex cord differentiation (11, 12). These tumors are characterized by their striking morphological resemblance to ovarian sex cord tumors, exhibiting a diverse array of architectural patterns including cords, trabeculae, nests, tubules, and retiform structures (13–15). The World Health Organization classification categorizes UTROSCTs as endometrial stromal and related tumors, recognizing their distinct clinicopathological features (16, 17).

The diagnostic complexity of UTROSCTs stems from their morphological overlap with various benign and malignant uterine neoplasms, including endometrial stromal tumors, leiomyomas with unusual patterns, epithelioid smooth muscle tumors, and even carcinomas (18–20). This diagnostic challenge is compounded by the limited value of preoperative imaging, as ultrasound and MRI findings often suggest more common entities such as uterine leiomyomas or adenomyosis (2, 21, 22). The preoperative biopsy interpretation is frequently inconclusive due to the tumor’s heterogeneous composition and sampling limitations (2, 9). Recent advances in molecular pathology have begun to illuminate the genetic landscape of UTROSCTs, with the discovery of recurrent gene fusions involving NCOA1-3, ESR1, and GREB1 genes (13, 23, 24). These molecular alterations not only provide diagnostic markers but also offer insights into the tumorigenesis of these rare neoplasms. Furthermore, emerging evidence suggests that specific genetic alterations may correlate with clinical behavior, potentially enabling better prognostic stratification (13, 14, 25).

The therapeutic approach to UTROSCTs has traditionally involved hysterectomy with or without bilateral salpingo-oophorectomy, reflecting the uncertainty regarding their malignant potential (9, 26, 27). However, growing recognition of their generally indolent behavior, particularly in younger women, has led to increased consideration of fertility-sparing approaches in selected cases (26, 28, 29). The management dilemma is further complicated by the occasional aggressive cases that demonstrate recurrence or metastasis, sometimes after prolonged disease-free intervals (6, 30, 31).

This article presents the diagnostic and therapeutic course of a patient with UTROSCT, reviews the literature regarding the clinicopathological characteristics, diagnostic challenges, molecular genetics, treatment strategies, and prognostic implications of this rare disease, aiming to provide an updated perspective and to highlight areas that warrant further research.

## 2 Case presentation

In April 2024, an otherwise healthy 38-year-old woman with no pertinent medical history presented to our institution with a six-month history of heavy menstrual bleeding (menorrhagia) and prolonged periods. Transvaginal ultrasonography showed an anteverted uterus with regular contours and a 3-mm endometrial stripe; both ovaries appeared normal. A well-circumscribed, oval, intramural mass—26 × 20 × 21 mm—bulging into the endometrial cavity was identified in the posterior uterine wall, with prominent intra-tumoral blood flow, consistent with a submucosal fibroid; no pelvic fluid was seen. After full discussion and informed consent, hysteroscopic resection was performed, achieving complete excision without macroscopic residual disease. The specimen was tan-white, firm, and grossly indistinguishable from a typical leiomyoma. The procedure was uneventful, and the patient was discharged home the next day.

**Figure 1:**
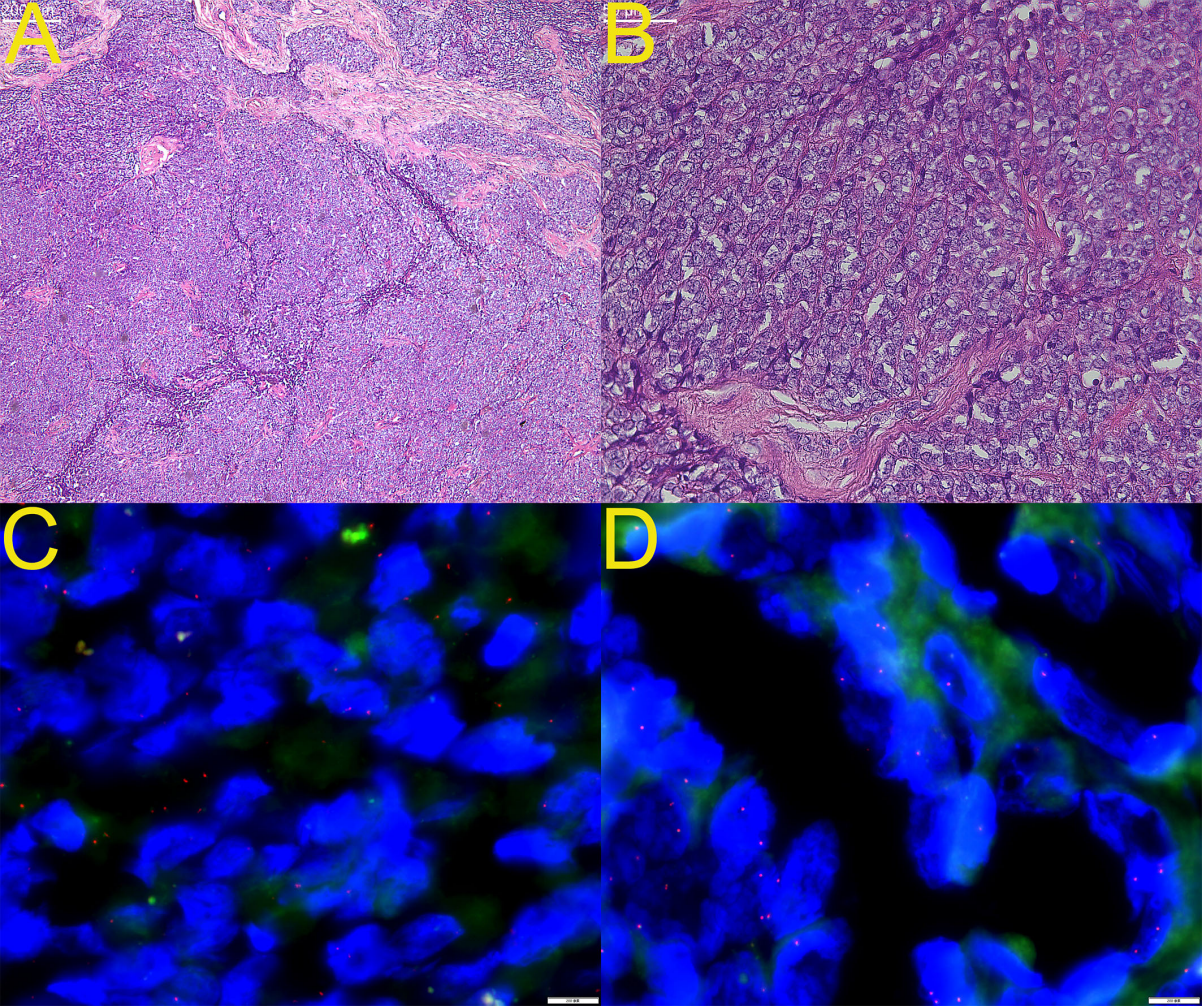
Histology and fluorescence *in-situ* hybridization (FISH) findings of the index uterine tumor resembling ovarian sex-cord tumor (UTROSCT). **(A)** Hematoxylin and eosin (H&E) stain at ×40 magnification shows anastomosing cords and trabeculae set in a richly vascular, mildly myxoid stroma. **(B)** Higher-power view (H&E, ×200) demonstrates monotonous oval-to-spindle cells with scant eosinophilic cytoplasm, smooth nuclear membranes, inconspicuous nucleoli, and mitotic figures ≤ 1/10 high-power fields; no necrosis or hemorrhage is present. **(C)** GREB1 break-apart FISH reveals intact fusion signals in all evaluable nuclei, indicating absence of GREB1 rearrangement. **(D)** ESR1 break-apart FISH likewise shows retained fusion signals, confirming lack of ESR1 translocation.

Microscopically, low-power examination revealed anastomosing cords, trabeculae, and occasional hollow tubules set in a richly vascular, mildly myxoid stroma (**Figure 1A**, ×40). High-power views showed a monotonous population of spindled-to-ovoid cells with scant eosinophilic cytoplasm, smooth nuclear membranes, inconspicuous nucleoli, and mitotic figures numbering 0–1 per 10 HPF; no necrosis or hemorrhage was present (**Figure 1B**, ×200). Immunohistochemical findings are summarized in **Table 1**: AE1/3 was diffusely positive, with positive staining for CD56 and WT1 and focal positivity for CD10; inhibin-α, calretinin, CD99, and EMA were negative. Entrapped smooth-muscle bundles demonstrated positivity for desmin and HHF-35. The Ki-67 proliferation index was approximately 5%. FISH analysis revealed no rearrangement of GREB1 (**Figure 1C**) or ESR1 (**Figure 1D**). Next-generation sequencing using a 141-gene solid tumor panel failed to identify pathogenic fusions involving ESR1, GREB1, NCOA2/3, or NR4A3, and showed no mutations in TP53, BRCA1/2, or mismatch-repair genes, consistent with the fusion-negative subset of UTROSCT seen in approximately 30–45% of cases. Taken together, the final diagnosis was “UTROSCT, fusion-negative subtype, low malignant potential, with negative resection margins.”

**Table 1:** Immunohistochemistry profile and interpretation.

Marker	Result	Interpretation	Diagnostic implication in present case
Pan-cytokeratin (AE1/3)	Diffuse +	Epithelial/sex-cord differentiation	Supports sex-cord-like phenotype
EMA	–	Excludes true epithelial neoplasm	Consistent with UTROSCT
CK18	–	Excludes glandular epithelium	Consistent
Sex-cord–stromal panel			
α-Inhibin	–	Classic sex-cord marker	Negative in ≈30–40% of UTROSCT; does not rule out diagnosis
Calretinin	–	Sex-cord/mesothelial	Same as above
FOXL2	–	Granulosa-cell differentiation	Negative
WT1	+	Sex-cord/renal blastema	Supports sex-cord lineage
CD99	–	Sex-cord/Ewing family	Negative
Neuro-endocrine			
CD56	Diffuse +	Neuro-endocrine–sex-cord overlap	Frequently positive in UTROSCT
Synaptophysin	+++	Neuro-endocrine vesicles	Expression compatible with UTROSCT
Chromogranin A	–	Neuro-endocrine granules	Excludes classic NET
NSE	–	Non-specific neuro-endocrine	Negative
Myogenic (entrapped muscle)			
Desmin	Focal +	Smooth-muscle differentiation	Represents residual myometrium
HHF-35 (muscle-actin)	Focal +	Smooth-muscle	Same as above
Miscellaneous/controls			
CD10	Focal +	Endometrial stroma	May be weakly positive; not diagnostic
ER	–	Estrogen receptor	Negative (≈30% of UTROSCT are ER-negative)
PR	–	Progesterone receptor	Same as above
BRG-1 (SMARCA4)	+++	SWI/SNF intact	Excludes SMARCA4-deficient tumor
INI-1 (SMARCB1)	+++	Retained expression	Excludes malignant rhabdoid tumor
Ki-67 index	≈5%	Proliferation index	low malignant potential
S-100	–	Neural sheath/melanocytic	Excludes schwannoma, etc.
Pan-TRK	–	NTRK fusion control	Excludes NTRK-rearranged spindle-cell neoplasm

A contrast-enhanced computed tomography scan of the pelvic performed 6 months postoperatively showed no residual disease or metastasis. Given the patient’s young age, desire for future fertility, and the tumor’s low-grade nature, hysterectomy with bilateral salpingo-oophorectomy was deferred. Twenty months of follow-up have since elapsed; the patient remains menstrually regular, reports no abnormal bleeding, and serial imaging shows no evidence of recurrence.

## 3 Discussion

### 3.1 Clinical pathological features

UTROSCTs typically present as well-circumscribed, solitary masses with a mean size of approximately 6 cm, though tumors ranging from 1 to 24 cm have been reported (8, 9, 27). These neoplasms most commonly arise in the uterine corpus as intramural lesions, but may also occur in submucosal, subserosal, or polypoid/intracavitary locations (3, 21). Less frequently, UTROSCTs involve the cervix, presenting exceptional diagnostic challenges due to their rarity in this location (32, 33). Gross examination typically reveals tan to yellow cut surfaces with a soft, fleshy consistency, sometimes with areas of degeneration, hemorrhage, or necrosis (8, 34, 35). Microscopically, UTROSCTs exhibit a remarkable diversity of architectural patterns, often occurring in combination within the same tumor (13, 14). The most characteristic patterns include anastomosing cords, trabeculae, nests, tubules, and retiform structures reminiscent of ovarian sex cord tumors (13, 15). Additional patterns such as diffuse sheets, fascicular arrangements, whorled formations, and microfollicular structures are also commonly observed (13, 14). The tumor cells are typically uniform with round to ovoid nuclei, fine chromatin, inconspicuous nucleoli, and moderate amounts of eosinophilic cytoplasm (15, 33). Nuclear grooves are rarely observed, unlike in ovarian granulosa cell tumors (15).

The immunohistochemical profile of UTROSCTs is notably polyphenotypic, often demonstrating coexpression of sex cord, epithelial, smooth muscle, and steroid hormone markers (3, 36, 37). A comprehensive meta-analysis of 181 UTROSCTs revealed the following pooled positivity rates for various markers: CD56 (97%), progesterone receptor (91%), estrogen receptor (85.5%), WT1 (84%), wide-spectrum cytokeratins (78.7%), CD99 (77%), desmin (74.5%), calretinin (70.6%), smooth muscle actin (56.4%), inhibin (44.5%), CD10 (41%), caldesmon (21.9%), and Melan-A/MART-1 (10.4%) (36).

### 3.2 Diagnosis and differential diagnosis

The diagnosis of UTROSCTs poses significant challenges due to their morphological diversity and immunohistochemical overlap with numerous other uterine neoplasms. The diagnostic process typically begins with imaging studies, although these often provide non-specific findings. Ultrasound examination frequently suggests uterine leiomyomas or adenomyosis, while MRI may show variable signal intensity on T1- and T2-weighted images, sometimes with heterogeneous enhancement (2, 21, 22). These non-specific imaging characteristics underscore the necessity of histopathological examination for definitive diagnosis (2, 21). Histological diagnosis relies on the identification of characteristic sex cord-like patterns, including cords, trabeculae, nests, and tubules, in the absence of a recognizable endometrial stromal component (3, 19). However, the extensive sampling is often required to appreciate the full spectrum of morphological features and to exclude other entities (20, 38). The diagnostic challenges are particularly pronounced in small biopsy specimens, where the limited tissue may not capture the representative morphology (2, 39). As shown in **Table 1**, the immunohistochemical evaluation plays a crucial role in confirming the diagnosis of UTROSCT and excluding morphological mimics.

The differential diagnosis of UTROSCT encompasses a broad spectrum of uterine neoplasms. Endometrial stromal tumors with sex cord-like elements (ESTSCLEs) represent the most challenging distinction (19, 38, 40). Unlike UTROSCTs, ESTSCLEs contain a conspicuous component of conventional endometrial stroma, typically comprising more than 50% of the tumor (19, 38). Additionally, ESTSCLEs often demonstrate tongue-like infiltration, spiral arteriole-like vessels, and frequent CD10 positivity (38, 40). Molecular studies can be helpful, as ESTSCLEs may harbor JAZF1-SUZ12 or other endometrial stromal sarcoma-associated fusions, while UTROSCTs typically feature NCOA1–3 rearrangements (24, 41, 42). Epithelioid smooth muscle tumors, particularly plexiform leiomyomas and leiomyosarcomas, can mimic UTROSCTs (43, 44). These tumors typically show stronger and more diffuse expression of smooth muscle markers (SMA, desmin, caldesmon, h-caldesmon) and are usually negative for sex cord markers (37, 44). However, the distinction can be challenging since UTROSCTs frequently express smooth muscle markers (36, 37). The presence of conventional areas of smooth muscle differentiation and the absence of sex cord marker expression favor smooth muscle tumors (44). Perivascular epithelioid cell tumors (PEComas) may enter the differential diagnosis, particularly those with nested or trabecular patterns (32, 45). PEComas typically express melanocytic markers (HMB45, Melan-A) and smooth muscle markers, but lack the broad polyphenotypic profile of UTROSCTs (37, 45). Additionally, PEComas often demonstrate distinctive perivascular epithelioid cell morphology and may harbor TSC1/TSC2 mutations (45). Uterine carcinomas with sex cord-like patterns, including sertoliform endometrioid carcinoma and corded/hyalinized endometrioid carcinoma, must be considered (19). These tumors typically show more overt epithelial differentiation, with gland formation, squamous metaplasia, or areas of conventional endometrioid carcinoma (19). They also demonstrate stronger and more diffuse epithelial marker expression and are usually negative for sex cord markers (19). Metastatic tumors to the uterus, particularly metastatic sex cord-stromal tumors from the ovary, should be excluded through clinical correlation and imaging studies (46). Primary ovarian sex cord-stromal tumors secondarily involving the uterus are rare but can present diagnostic difficulties (46).

### 3.3 Molecular genetic features

The molecular landscape of UTROSCTs has been increasingly elucidated in recent years, revealing recurrent genetic alterations that provide insights into their pathogenesis and offer diagnostic markers (13, 24, 41). The most characteristic molecular alterations in UTROSCTs are gene fusions involving NCOA1, NCOA2, or NCOA3, which serve as the 3’ partners, with various 5’ partners including ESR1, GREB1, and less commonly CITED2 or other genes (13, 23, 24). These discoveries have fundamentally advanced our understanding of UTROSCT biology and provided molecular criteria for diagnosis. NCOA1–3 rearrangements are detected in approximately 70-80% of UTROSCTs, with variations in reported frequencies across different studies (13, 24, 41). The most common fusion partners are ESR1 and GREB1, with ESR1-NCOA3 being the most frequent fusion type, followed by GREB1-NCOA2, ESR1-NCOA2, and GREB1-NCOA1 (13, 41). Less common fusions include GREB1-NCOA3, ESR1-CITED2, GREB1-CTNNB1, and GREB1-NR4A3 (1, 6, 47). These fusions are mutually exclusive and appear to define molecular subgroups with distinct clinicopathological characteristics (13, 14). Emerging evidence suggests correlations between specific fusion types and clinicopathological features. GREB1-rearranged UTROSCTs tend to occur in older patients, present as intramural masses, and demonstrate more aggressive behavior with higher recurrence rates (13, 14, 48). In contrast, ESR1-rearranged tumors more frequently present as polypoid or submucosal masses and generally follow a more indolent course (13, 14). GREB1-NCOA2 fusions in particular have been associated with aggressive behavior, with recurrence rates approaching 57% in some series (14, 49). Tumors with ESR1-CITED2 fusions have also demonstrated aggressive potential, including recurrence and metastasis (6, 23). The functional consequences of these fusion events are beginning to be understood. ESR1 and GREB1 are both estrogen-responsive genes involved in hormone signaling pathways, while NCOA1–3 encode nuclear receptor coactivators that modulate transcriptional activity (24, 50). The fusion proteins are believed to constitutively activate estrogen-responsive pathways, potentially explaining the hormone receptor positivity observed in most UTROSCTs (24, 50). The GREB1-CTNNB1 fusion is particularly interesting as it results in nuclear accumulation of β-catenin and constitutive activation of Wnt signaling, providing an alternative oncogenic mechanism (47). The molecular heterogeneity of UTROSCTs extends beyond the recognized fusion types. Some tumors lack detectable NCOA1–3 rearrangements despite typical morphological features, suggesting alternative genetic mechanisms (13, 41). These fusion-negative cases may represent technical limitations or truly distinct biological entities requiring further characterization (13). Additionally, the relationship between UTROSCTs and other uterine sarcomas with similar genetic alterations, such as so-called “GREB1-rearranged uterine sarcomas,” remains to be fully clarified (48, 50).

### 3.4 Treatment strategies and management

The management of UTROSCTs presents therapeutic challenges due to their rarity, variable clinical behavior, and the lack of prospective studies to guide treatment decisions (9, 26, 27). The historical standard treatment has been total hysterectomy with or without bilateral salpingo-oophorectomy, reflecting the uncertain malignant potential of these tumors (9, 26, 27). However, evolving understanding of their generally indolent behavior, particularly in molecularly defined subgroups, has led to more individualized approaches that consider patient age, fertility desires, and tumor characteristics (26, 28, 29). For women who have completed childbearing or are perimenopausal/postmenopausal, total hysterectomy remains the treatment of choice (2, 26, 27). The role of bilateral salpingo-oophorectomy is debated, as UTROSCTs are uterine primaries without demonstrated ovarian tropism (26, 27). A systematic review of 147 patients found no significant difference in 10-year disease-free survival between patients who underwent hysterectomy alone versus those who also had bilateral salpingo-oophorectomy (26). Lymphadenectomy is not routinely recommended unless there is evidence of lymph node involvement or other high-risk features (9, 35). Fertility-sparing approaches have gained acceptance for young women who wish to preserve reproductive capacity (26, 28, 29). Conservative surgery typically involves tumor resection via hysteroscopy or laparoscopy, with the goal of complete excision with negative margins (28, 29, 51). A comprehensive review found that among 15 patients under 40 years of age who underwent mass resection, 7 (46.7%) achieved pregnancy and 6 (40%) had successful deliveries (26). The recurrence rate after fertility-sparing surgery was 30%, compared to 17.6% after hysterectomy (26). However, no significant differences in 5- and 10-year disease-free survival were observed between the two approaches (26). The selection of patients for conservative management should be based on careful consideration of tumor characteristics. Favorable features include small tumor size, well-circumscribed borders, low-grade cytology, low mitotic activity, absence of necrosis or lymphovascular invasion, and possibly ESR1 rearrangements (7, 26, 29). Patients should be counseled about the risk of recurrence and the recommendation to proceed with definitive surgery after completing childbearing (26, 29). Close follow-up with regular imaging, preferably MRI, is essential (8, 29). Studies have shown that UTROSCTs harboring GREB1 rearrangements—particularly the GREB1–NCOA2 fusion—are associated with more aggressive behavior and higher recurrence rates (13, 14, 48, 49). In the present case, next-generation sequencing failed to identify pathogenic fusions involving ESR1, GREB1, NCOA2/3, or NR4A3, placing the patient in a molecular subset linked to lower aggressiveness and a reduced probability of relapse. This finding, combined with the patient’s nulliparous status, underpinned the multidisciplinary team’s decision to defer definitive hysterectomy and continue fertility-preserving surveillance in this young woman.

Post-treatment surveillance recommendations are based on limited evidence but generally include regular clinical examination and imaging (8, 29). MRI is preferred over ultrasound for follow-up due to its superior soft tissue resolution and ability to detect deep myometrial involvement or recurrence (8, 21). The optimal duration of follow-up is uncertain given the potential for late recurrences, sometimes occurring decades after initial diagnosis (30, 31). Long-term surveillance is recommended, particularly for cases with high-risk features (7, 31). Multidisciplinary management involving gynecologic oncologists, pathologists, radiologists, and medical oncologists is essential for optimal care of UTROSCT patients (9, 35). Tumor boards can help review difficult cases, particularly those with atypical features or when conservative management is considered (9, 35). Participation in registries and clinical trials, when available, is encouraged to advance understanding of these rare tumors (7, 52).

### 3.5 Prognosis and recurrence

UTROSCTs are generally considered to have low malignant potential, but a subset demonstrates aggressive behavior with recurrence and metastasis (7, 27, 31). The overall recurrence rate ranges from 5% to 30% across different studies, with metastatic rates of approximately 7% (26, 27, 31). Recurrences can occur locally in the pelvis or as distant metastases to sites including lungs, liver, bone, abdominal and pelvic peritoneum, lymph nodes, and omentum (31, 53, 54). The time to recurrence is variable, ranging from 9 months to 32 years after initial diagnosis, emphasizing the potential for late recurrences and the need for long-term follow-up (30, 31, 55).

Despite the potential for recurrence, the overall survival of UTROSCT patients is generally favorable (26, 27). Disease-specific death is rare, occurring in approximately 3-9% of cases (27, 31). The 5-year disease-free survival rates range from 70% to 97% across different studies (26, 27, 55). The wide variation in reported outcomes reflects differences in study populations, follow-up duration, and possibly evolving diagnostic criteria over time (7, 27, 31). The management of recurrent disease is challenging due to the limited number of cases and absence of standardized guidelines (53, 54). Surgical resection of recurrent lesions, when feasible, is generally recommended (53, 54). Systemic therapies including chemotherapy and hormonal agents have been used with variable success (2, 53). Radiation therapy has been employed in some cases, particularly for local control (2). The response to treatment is unpredictable, and some patients experience indolent courses even with metastatic disease (31, 54).

Long-term follow-up is essential given the potential for late recurrences (30, 31). Surveillance typically includes regular clinical examinations and imaging studies, with MRI being the preferred modality for detecting local recurrence (8, 21). The optimal frequency and duration of follow-up are not established, but given the documented cases of very late recurrence, extended surveillance is prudent (30, 31). Patient education about symptoms of recurrence is an important component of long-term management. Prognostic stratification remains challenging due to the rarity of UTROSCTs and the limited predictive value of individual histological features (7, 31). Although our patient remains disease-free at 20 months, UTROSCT can recur even decades after primary surgery and that life-long surveillance is therefore recommended, especially when fertility-sparing resection is performed. Integrated models incorporating clinical, pathological, immunohistochemical, and molecular parameters may improve risk assessment in the future (13, 14, 25). The development of such models will require multi-institutional collaboration and prospective data collection (7, 52).

## 4 Conclusion and future perspectives

In conclusion, UTROSCTs remain a diagnostic and therapeutic challenge, but recent molecular advances have provided new insights into their biology and tools for improved diagnosis. A multidisciplinary approach incorporating clinical, pathological, and molecular information is essential for optimal management. Future research efforts should focus on refining risk stratification, developing targeted therapies, and establishing evidence-based guidelines through collaborative studies.

## Data Availability

The original contributions presented in the study are included in the article/supplementary material. Further inquiries can be directed to the corresponding author.
